# Adoptive cell transfer: new perspective treatment in veterinary oncology

**DOI:** 10.1186/s13028-018-0414-4

**Published:** 2018-10-11

**Authors:** Joanna Katarzyna Bujak, Rafał Pingwara, Michelle Hase Nelson, Kinga Majchrzak

**Affiliations:** 1 Department of Physiological Sciences, Faculty of Veterinary Medicine, Warsaw University of Life Sciences, ul. Nowoursynowska 159, 02-776 Warsaw, Poland; 2 Department of Microbiology and Immunology, Medical University of South Carolina, 86 Jonathan Lucas Street HCC-612, Charleston, SC 29425 USA

**Keywords:** Adoptive cell transfer, Canine oncology, Gene editing, Immunotherapy, T lymphocytes

## Abstract

Cancer immunotherapy is recently considered the most promising treatment for human patients with advanced tumors and could be effectively combined with conventional therapies such as chemotherapy or radiotherapy. Patients with hematological malignancies and melanoma have benefited greatly from immunotherapies such as, adoptive cell transfer therapy, experiencing durable remissions and prolonged survival. In the face of increasing enthusiasm for immunotherapy, particularly for the administration of tumor-specific T lymphocytes, the question arises whether this method could be employed to improve treatment outcomes for canine patients. It is warranted to determine whether veterinary clinical trials could support comparative oncology research and thus facilitate the development of new cell-based therapies for humans. Herein, we discuss adoptive transfer of T lymphocytes and lymphokine-activated cells for application in veterinary oncology, in the context of human medicine achievements. Furthermore, we discuss potential benefits of using domestic dog as a model for immunotherapy and its advantages for translational medicine. We also focus on an emerging genome-editing technology as a useful tool to improve a T cells’ phenotype.

## Background

Cancer is a complex disease caused by the impairment in a cells’ physiology leading to uncontrolled proliferation and inhibition of apoptosis [1]. Disease progression results from a complicated interplay between genetic alterations of transformed cells and cancer immunoediting by the hosts immune defense mechanisms [2]. It has been indicated in multiple human and canine studies that the dysfunction of immune system, enabling tumor growth and metastasis, is associated with tumor immune escape. This process is mainly manifested by downregulated expression of major histocompatibility complex (MHC) class I and tumor specific antigens, as well as, by production of anti-inflammatory cytokines such as IL-10 and TGF-β by malignant cells [3, 4]. Local immunosuppression is further supported by active recruitment of myeloid-derived suppressor cells (MDSC) into tumor microenvironment and activation of suppressive T regulatory cells (Tregs). This unfavorable niche alters the fate of immune cells and contributes to the functional inhibition of effector T and NK cells (Natural Killer cells), resulting in immunologic tolerance [5]. Unresponsiveness of T cells is caused by chronic stimulation and the expression of co-inhibitory receptors such as Programmed cell death protein 1 (PD-1) and cytotoxic T cell antigen 4 (CTLA-4), which leads to T cell exhaustion [6]. Moreover, cancer cells can induce deactivation of circulating monocytes and polarization of macrophages to M2-like phenotype, which not only foster existing tumor but also facilitate spread of transformed cells [7, 8]. Promotion of cancer progression is also linked with production of pro-angiogenic and pro-metastatic factors by tumor-associated macrophages (TAMs) and MDSCs [8–10]. Given the complex and dynamic crosstalk within the tumor microenvironment, the development of an effective anticancer immunotherapy has been a challenging endeavor.

The first report of ACT therapy date back to mid-1960’s, when allogeneic T lymphocytes have been transferred into rats to treat primary fibrosarcoma [11]. The goal of the study was to harness cytotoxic CD8^+^ T cells (CTLs), capable of mediating direct target cell lysis, to fight against cancer. These landmark experiments paved the way for the development of cellular immunotherapy. Further advances have resulted in the discovery of cancer-associated antigens and the improvement of genetic engineering.

Currently, ACT therapy has demonstrated great promise in eliciting curative responses against hematological malignancies and melanoma in human patients. Veterinary oncology is highly translatable for human medicine and results obtained in the canine patients can facilitate the design of the next-generation clinical trials to treat advanced solid tumors in humans.

## Search strategy

This review is based on a search in PubMed (http://www.ncbi.nlm.nih.gov/pubmed) using the terms “adoptive cell transfer” OR “adoptive cell transfer in dogs” AND “tumor infiltrating lymphocytes” OR “TILs” AND “TCR engineered T cells” AND “CAR T cells” OR “canine CAR T cells” AND “canine T-LAK” AND “genome editing” OR “genome editing therapy”. Only papers written in English were included in the review. The vast majority of the literature cited, is less than 15 years old. Exceptions are the papers that describe for the first time the crucial method or discovered phenomenon in the field of immunotherapy (i.e. first studies that paved the way for immunotherapy as a historical link).

All original research related to the canine immunotherapy (more specifically canine adoptive cell transfer and T-LAK therapy) were incorporated. Studies related to adoptive cell immunotherapy and genome editing, were evaluated and the most relevant to the review were selected. Our systematic review comprises the current knowledge on adoptive cell transfer therapy in canine oncology, in the context of human medicine achievements.

## Advantages of using a dog model for comparative oncology

The domestic dog (*Canis lupus familiaris*) is an attractive and useful model for comparative medicine for the evaluation and development of novel therapeutic strategies and ensuing immunological assessments [12–16]. Unlike transplantable xenograft rodent models, canine tumors share with human tumors similar epidemiology, genetic, biology, treatment responses, prognosis factors and clinical outcomes. Cancer occurs in dogs naturally and spontaneously and the progression of disease (e.g. metastatic cascade) is similar to humans. Finally, dogs share the same life environment and thus cancerogenic and risk factors with people [17]. Similar to people, the incidence of cancer in dogs increases with age, with a frequency of 45% in animals older than 10 years [18]. Therefore, the domestic dog is a recommended model for multiple human diseases including cancers such as non-Hodgkin lymphoma, osteosarcoma, leukemia, melanoma and lung, head and neck, prostate, mammary, and bladder carcinomas [12, 19, 20].

Cancer research has been revolutionized through the advances in genome sequencing and assembly. Knowledge about the genetic basis and molecular mechanisms of cancer progression greatly accelerated the development of novel therapies. In 2005, the canine genome was sequenced, leading to more advanced studies in the field of veterinary sciences [21]. It was an important step for comparative studies that improved the understanding of mammalian evolution as well as enhanced our knowledge of tumorigenesis, cancer growth and metastasis, and tumor immunology in animals.

**Fig. 1:**
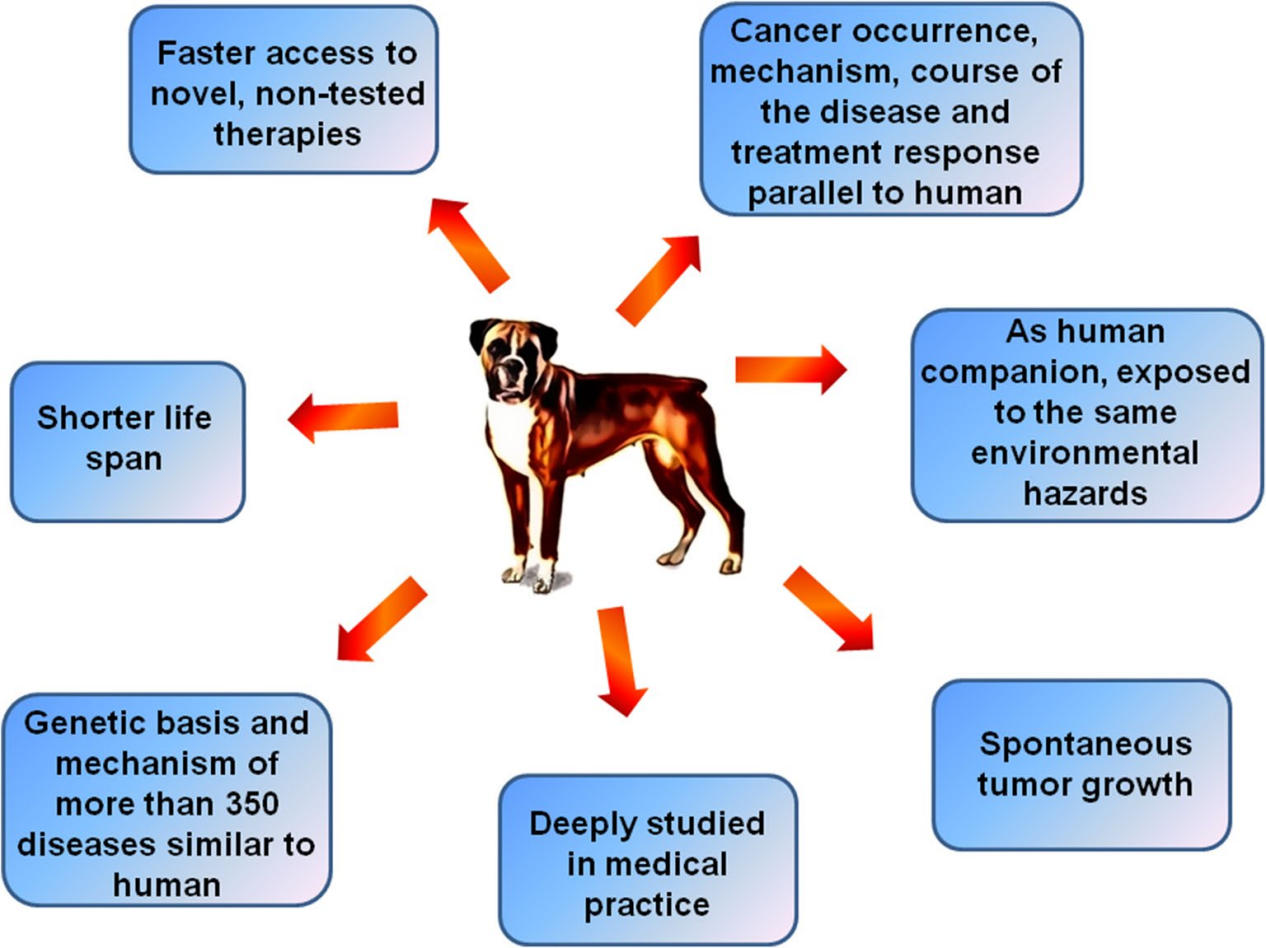
The domestic dog can serve as an attractive model in comparative oncology. Dogs and humans share the same environment and possess multiple similarities in genetics, physiology as well as tumorigenesis and cancer progression. For this reason, canine models are of great importance to cancer and immunological studies and can contribute to improvement of human immunotherapy

The innate and adaptive immune system in dogs is comparable to humans. Additionally, the main immune cells subsets identified in dogs, as well as their surface markers, exhibit similarities to human [16]. Although many similarities have been found our understanding of canine immune system functioning is still highly affected by lack of specific reagent to fully define all immune subsets. Furthermore, factors regulating immune response and subsequent signaling pathways responsible for induction of anticancer immunity are yet to be determined. Nevertheless, domestic dog model offer possibilities of clinical studies that will benefit and inform design of human clinical trials. Advantages of using canine animal models are summarized in Fig. 1.

## Adoptive T cell transfer therapy

Among the four different categories of immunotherapies including: checkpoint inhibitors, monoclonal antibodies, therapeutic vaccines and cellular therapies, it is adoptive T cells transfer (ACT) therapy that has recently shown clinical outcomes of record efficiency in human hematological malignances and melanoma [22, 23].

ACT treatment involves the infusion of tumor-specific T lymphocytes into the circulatory system of cancer patients. The immune cells can be derived from one of two sources: (1) natural host T cells identified in the tumor mass—the autologous tumor infiltrating lymphocytes (TILs), (2) autologous T cells from patients’ peripheral blood that have been genetically engineered ex vivo to express specific antitumor T cell receptors (TCRs) or chimeric antigen receptors (CARs) [24, 25]. The cells obtained from patients are activated and extensively expanded to large numbers (1 × 10^11^) ex vivo in the presence of high dose IL-2 (a T cell growth factor) before re-infusion. Recently, additional genome editing has been proposed to enhance the function of engineered T lymphocytes.

**Fig. 2:**
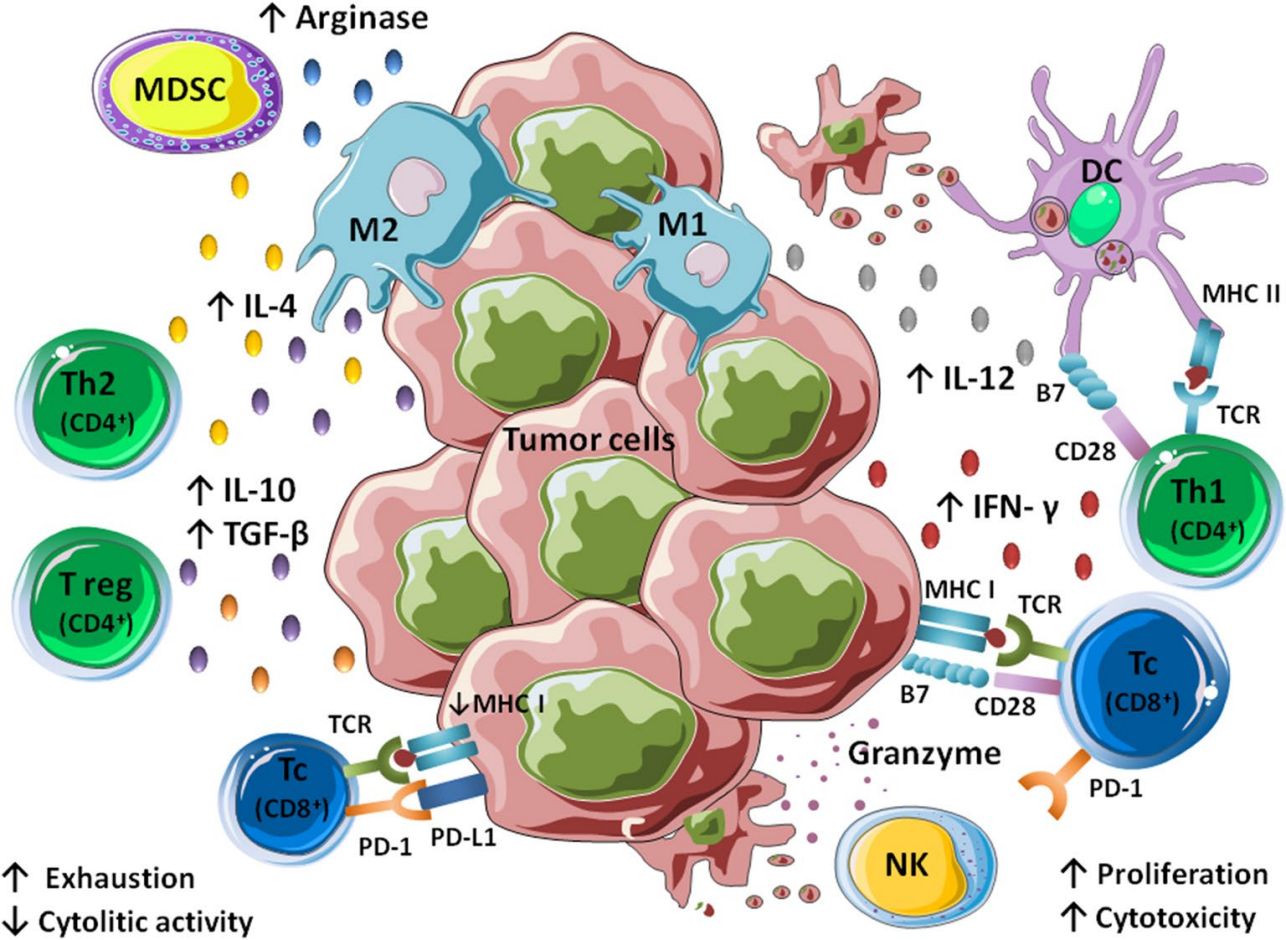
Tumor microenvironment consists of malignant cells, stroma and different populations of immune cells. Complex crosstalk between them shapes the final outcome of neoplastic disease. Anticancer response is driven mainly by cytotoxic CD8^+^ T cells and NK cells, which release IFN-γ and granzymes, thus are involved in direct lysis of the tumor cells. Th1 subpopulation of CD4^+^ T cells, M1 macrophages and activated dendritic cells (DCs) support anticancer immunity by antigen presentation and cytokine production (IL-12, IFN-γ). CD8^+^ and CD4^+^ T cells recognize tumor antigens in the context of MHC class I and II respectively, followed by costimulatory signaling via CD28 molecule, necessary for their full activation, proliferation and function. Tumor progression, in turn, is associated with the presence of the Th2 and T regulatory CD4^+^ lymphocytes, M2 macrophages and MDSC. These cells secrete immunosuppressive factors such as IL-4, IL-10, or TGF-β and exhibit high activity of arginase, respectively. Unresponsiveness of cytotoxic CD8^+^ T cells is caused by decreased expression of MHC I on the cancer cells surface and activation of coinhibitory receptors such as PD-1. Adapted from Servier Medical Art

In veterinary medicine, due to the similarities between canine and human antitumor immunity, a parallel approach can be applied (Fig. 2). Herein, we will compare ACT therapy in human versus veterinary settings.

## Tumor infiltrating T lymphocytes

**Fig. 3:**
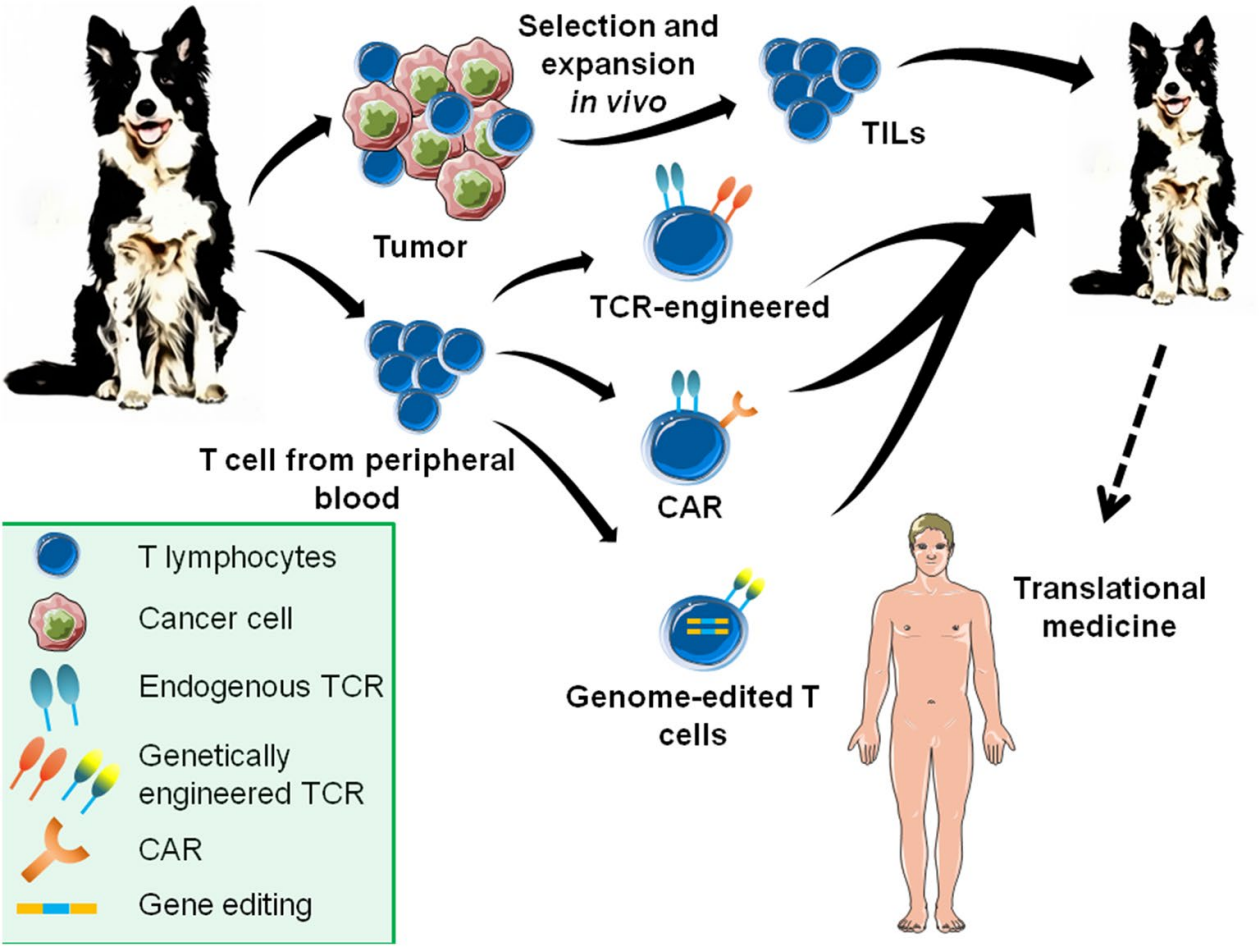
Adoptive cell immunotherapy possibilities for cancer in dogs include transfer of TILs, TRC-modified and CAR-engineered T lymphocytes. Tumor reactive T cells can be derived from tumor mass or peripheral blood lymphocytes can be genetically engineered to recognize tumor-specific antigens. Obtained T cells are expanded ex vivo and then administrated back to the tumor-bearing patient. Recently, genome editing technologies are using to confer additional modifications to T cells such as disruption of endogenous TCR and MHC. Introduction of ACT treatment into veterinary medicine can greatly facilitate the design of the new clinical trials for humans. Adapted from Servier Medical Art

The tumor microenvironment consists of transformed cancer cells and non-transformed stromal and inflammatory cells including fibroblasts, endothelial cells, T lymphocytes, dendritic cells, MDSCs and macrophages. Dynamic crosstalk between all of them via released cytokines and growth factors, and direct cell–cell interactions is responsible for either promotion or inhibition of tumor growth and metastasis (Fig. 3).

Among the immune cells that penetrate a tumor, the tumor infiltrating lymphocytes (TILs), are capable of mediating cancer cells eradication. Increased T cell infiltration is a good prognostic marker for multiple solid tumors including, breast cancer (reviewed: [26, 27]) head and neck squamous cell carcinoma [28], melanoma [29, 30], muscle-invasive bladder cancer [31], seminoma [32], pancreatic adenocarcinoma [33], cervical [34], ovarian [35], gastric [36] and colorectal [37] tumors.

Most of the TILs in the tumor environment, due to the immunoediting process, experience chronic activation and exhaustion leading to the attenuation of proliferation and effector functions via activation of checkpoint co-inhibitory receptors such as PD-1 or CTLA-4 [6]. In some solid tumors, TILs are present in too low frequency to mediate a potent immune response [38]. Thus, TILs are retrieving from tumor mass, expanded ex vivo and re-infused back to the cancer patients. Tumor-specificity of TILs is selected based on IFN-γ production while co-cultured with cancer cells. Rosenberg et al. [39] were the first to demonstrate the regression of metastatic melanoma in patients that received autologous TILs along with high doses of IL-2 treatment.

However, after initial success with TIL therapy, further investigation revealed that transferred T cells do not persist long-term. In fact, infused T cells diminished over time and were undetectable in the blood of some cancer patients 2 weeks post-transfer, which resulted in a lack of objective responses [40]. The cytotoxic T cells used for transfer were comprised of terminally differentiated effector cells, without self-renewal capacity. Moreover, for CTLs to induce a potent antitumor response they depend on the activity of effector CD4^+^ T cells and the availability of homeostatic cytokines [41–43]. Thus, upon transfer into an immunocompetent recipient, CTLs were not able to survive and mount an effective response.

Preconditioning of cancer patients significantly improves the therapeutic efficiency of TIL therapy. This involves the systemic destruction of the recipient immune system (lymphodepletion) before ACT-based treatment. It was shown that chemotherapy consisting of high-dose of cyclophosphamide and fludarabine or total body irradiation of non-myeloablative (2 Gy) dose caused lymphodepletion, which significantly enhanced ACT efficiency [44, 45]. Such a regimen, prior to infusion, is necessary to destroy host immune cells and create ‘space’ for expansion of adoptively transferred T lymphocytes. The mechanism relies also on the elimination of immunosuppressive Tregs and MDSCs and by augmenting the innate immune system. Lymphodepletion excludes immune cells termed “cytokine sinks” that compete with infused T cells for homeostatic cytokines, thereby increasing access to those cytokines, particularly IL-15 and IL-7, which promote T lymphocyte proliferation in vivo [46].

Autologous TIL treatment was currently demonstrated to induce durable, complete, and in some cases curative metastatic melanoma regression with objective tumor response observed in around 50% of patients [47, 48]. Nevertheless, adaptation of this therapy for other types of solid tumor is limited due to low availability of TILs from tumor mass [49, 50]. To date, melanoma remains most immunogenic type of tumor and even patients in advanced stage IV of melanoma can benefit from autologous TIL transfer. However, still short-term persistence of infused T cells that are fully differentiated and exhausted constitutes an obstacle [51, 52]. Thus, extensive studies are ongoing to improve the protocols of ex vivo expansion and preparation of TILs (i.e. using pharmacological modifications). As of September 2018 there are 158 open clinical studies regarding administration of TILs to human cancer patients (http://www.clinicaltrials.gov). Advantages and disadvantages of using TILs are summarized in Table 1.

**Table 1:** The major advantages and disadvantages of various methods of generating T cells for the purpose of adoptive transfer

Therapy type	Advantages	Disadvantages
Tumor-infiltrating lymphocytes (TILs)	Recognize tumor-specific antigens	Isolation difficulties—not applicable for all of the cancer types
	High objective response against cancer reaching even up to 70%	Labor and time consuming
	Sustained remission	Not applicable for larger group of patients
	Low recurrence rate	Not all of the TILs are reactive to tumor antigens
		Presence of tumor-reactive T cells is indispensable
TCR-engineered lymphocytes	Does not require presence of tumor antigen-specific T cells	Competition between transgenic TCR and endogenous TCR
	Lymphocytes can be obtained from blood not from tumor tissue	Heterodimer formation and possibility of gaining unknown antigen-specificity
		Expression of two distinct TCR was associated with autoimmunity
Chimeric antigen receptor (CAR)—engineered T lymphocytes	Specific for broad-range of antigens including non-proteinaceous Ag	Fusion of various signaling domains may alter proper signaling cascade
	MHC-independent antigen recognition	CAR expression may not be stable
	Suitable for a relatively wide range of patients—not HLA-restricted	Uncertainties regarding the type of signaling domains and their order for proper T cell function
	Production of large quantities in relatively short time	Antigen specificity must be selected with caution
	Putative improvement of T cell properties such as proliferation, activation or cytokine secretion by insertion of specially designed CAR	May cause toxicity (on target/off tumor effect)
		CAR biology and interaction with different cancer types and TME not well defined yet
		Identification of optimal CAR still based on experimental procedures
Genome editing technologies (ZFN, TALENS, CRISPR/Cas)	Enhancement of T cells biology (e.g. resistance to TMI, cytokine secretion)	Possible off-target effect
	Applicable for wide-range of dysfunctions	Technical problems with delivery and efficiency
	Allow to overcome limitations of TCR-engineered and CAR T cells (elimination of endogenous TCR expression)	May induce immune response against bacterial components of GE technology
	Applicable for greater number of patients	Controversial, ethical issues

## TILs in veterinary oncology

Tumor infiltrating lymphocytes in canine cancers are not well characterized, therefore there is still much to be gleaned. An increase in T lymphocyte infiltration was correlated with spontaneous, rapid regression of transmissible venereal sarcoma [53], oral papilloma [54] and cutaneous histiocytoma [55].

Lymphocyte infiltration has been studied in canine mammary gland tumors as it is an attractive biological model for the investigation of human breast cancer immunology [56]. Initial studies showed an increase in infiltrating T cells in the tumors that gave metastasis, suggesting that TILs, especially CD4^+^ T cells promote tumor expansion [57, 58]. In contrast, the lab of Carvalho [59] demonstrated that malignant mammary tumors had a reduced number of intratumoral T cells compared to benign tumors. However, the proportion was reversed when CD3^+^ T cells were counted in the peripheral parts of the tumor or in the adjacent non-malignant mammary gland, indicating that T cells from that region might be engaged in tumor progression. In addition, an increase of TILs was associated with increased canine mammary tumor malignancy [60]. Unfortunately, the published data lacks in-depth T cell subset analysis, specifically the identification of Tregs, which are known to exhibit immunosuppressive properties thereby supporting cancer development [60].

Recent data confirms that a high frequency of T cells found within malignant tumors is due to a high percentage of Tregs [61]. Similarly, Mucha et al. [62] demonstrate an increased population of FoxP3^+^ (regulatory T cell-associated transcription factor) cells in canine malignant and metastatic mammary cancers compared to benign tumors. Likewise, Tominaga et al. [63] found, high Treg frequency in the peripheral blood and even higher in the tumor mass of dogs with oral melanoma.

Although, no studies have utilized TILs for ACT therapy in dogs with solid tumor, there is ongoing research which exploits T cells from canine patients with lymphoma [64]. In this case, however, the use of the term “TILs” can be speculative since in the hematological malignancies T cells do not truly infiltrate the tumor. T cells used in the following study were memory T cells which exhibit anticancer activity. These studies revealed that the transfer of autologous T lymphocytes from dogs with non-Hodgkin lymphoma, previously treated with chemotherapy, improved clinical outcomes. T cells isolated from the blood of lymphoma-bearing dogs were co-cultured with γ-irradiated K562 cells genetically modified to function as artificial antigen presenting cells (aAPC), in the presence of rhIL-21 and rhIL-2. Recently, this approach was also used to expand canine CAR T cells [65]. For the purpose of ACT, the majority of the cells were CD3^+^/CD8^+^ (88%). Importantly, 70% of the cells were memory T cells (CD3^+^, CCR7^+^) characterized by high production of IFN-γ. Dogs treated with chemotherapy and ACT therapy achieved complete remission lasting at least 338 days post T cell transfer. Overall percentage of CD8^+^ T cells was higher in the blood for up to 42 days of canine patients that received ACT therapy compared to their non-treated counterparts. The increase of CD8^+^ T cells was correlated with increased expression of thymidine kinase, enhanced granzyme B production and decreased neutrophil to lymphocyte ratio. The study demonstrated the successful application and clinical outcomes of tumor immunotherapy for canine patients. Of note, this was the first study to show the power of this effective therapy in in vivo study providing a solid foundation for further studies in the field of veterinary oncology.

## Utilizing T cell receptor transduction for ACT therapy

Antitumor activity of T cells is based on the ability of a T cells to recognize antigen via its endogenous T cell receptor (TCR). TCR is a transmembrane protein complex, consisting of α- and β-chains (more rarely δ- and γ-chains). Although, it was demonstrated that tumor-specific T cells can be successfully isolated from both tumor microenvironment and the blood for the purpose of ACT [66, 67], the limitations of this approach still hinder its wide application in medicine. The major drawback is related to accessibility of TILs in certain tumor types [50]. To overcome this limitation, the use of TCR-engineered T cells has been extensively studied.

The first studies, performed with mouse models, demonstrated that transfer of TCR α and β genes, targeted to tumor antigens, into T cells confers tumor antigen-specificity [68, 69]. These pioneering studies led to significant progress in the field of TCR-engineering for T cell therapy. To date, TCR-engineered T cells have been used against renal cell carcinoma antigen (RCC) [70], Wilms tumor antigen-1 (WT1) for leukemia cells [71], melanoma antigen recognized by T cells 1 (MART1) for patients suffering from melanoma [72] or cancer–testis antigen (NY-ESO-1) to treat myeloma [73]. Each of the studies showed the benefit with objective responses and potent antitumor activity.

The advantage of TCR-engineered T cells is that lymphocytes can be easily obtained from the peripheral blood and then transformed into tumor-reactive cells. Nevertheless, the major limitation of this method arises from off-target TCR heterodimer formation (Table 1). With the addition of a second α and β TCR chain, it is probable for the expression of four different TCR receptors due to interactions of endogenous and introduced α and β TCR chains [74]. Studies by Sommermeyer laboratory [75] have indicated that TCR with strong antigen affinity may replace TCR with weak antigen affinity, resulting in T cells with altered or novel antigen specificity. Furthermore, simultaneous expression of two distinct TCR was associated with an induction of autoimmunity [76–78].

Numerous clinical trials reveal potential toxicity of TCR-redirected T cell therapy, arising from on-target but off-tumor effects that in extreme cases lead to lethal outcomes [79]. For this reason, the crucial step in development of effective and safe treatment is selection of tumor-specific TCR.

TCR-engineered T cells have not been used in veterinary oncology primarily due to the fact that antigen recognition is based on MHC-restricted epitopes. Recently, however, Barth et al. [80] successfully identified a binding motif of canine MHC I allele DLA-88*50101. Interestingly, this binding motif exhibited high similarity to human leukocyte antigen HLA-A*02:01. Therefore, the authors stressed that canine models would be suitable for testing TCR-transduced T cell-based immunotherapies. Importantly, the canine genome has been sequenced which will help in the development of novel targets that can be implemented in both human and veterinary medicine.

## Chimeric antigen receptor (CAR) engineered T cells

The main restriction in using TCR-engineered T cells results from heterodimer formation resulting in undefined antigen specificity. Importantly, T cells can recognize antigen via its TCR only when it is processed into a peptide and presented in the context of an MHC molecule. However, malignant cells often downregulate expression of MHC molecules in order to avoid immune response [81]. Due to these limitations, researchers have developed a novel solution. The chimeric antigen receptor (CAR) fuses a single chain variable fragment (scFv) of an antibody that recognizes antigen with the intracellular signaling domains of the CD3ζ. The initial studies, conducted by Gross et al. [82], proved that T cells can successfully express chimeric receptors that recognize specific antigens and trigger T cell response. To date, three generations of CARs can be distinguished, based on the signaling endodomains [83]. First generation CARs consist solely of an antigen binding domain and the CD3ζ signaling domain necessary for T cell activation, while the 2nd generation incorporated a co-stimulatory signaling such as CD28 or inducible T cell costimulator (ICOS) [84]. Third generation of CARs combine additional co-stimulatory molecules to enhance the potency [85]. It was demonstrated that each generation of CARs exhibited distinctive properties with regard to the T cells’ antitumor activity, proliferation, cytotoxic properties, and persistence [86]. Studies by Hombach and Abken [87] have revealed significant differences, mainly related to proliferation and cytokine secretion, between lymphocytes expressing CARs recognizing the same epitope but with various costimulatory endodomains. Also, Bridgeman et al. [88] pointed out that results may differ significantly from each other even if the same chimeric receptors were used. Consequently, the design of artificial chimeric receptors requires further refinements and definition of the most optimal CAR structure.

There is great potential for adoptive therapy with CAR-engineered T cells as a treatment for some types of the cancer such as neuroblastoma and leukemia [89, 90], yet in other cancer types (e.g. ovarian cancer and metastatic renal cell carcinoma) the therapy have demonstrated poor responses [91, 92]. Additionally, these studies have documented on-target/off-tumor toxicities after CAR-T cell treatments. Nonetheless, it was proposed that on-target toxic effects of CAR-T cell therapy can be abolished by administration of agents that block specific epitopes, such as monoclonal antibodies [93].

Many aspects of CAR-T cell therapy require further investigation however, the therapy induce a powerful antitumor response. The latest trends in CAR-T cell research are designing the next-generation CAR-T cells that will be engineered with suicide genes or dual-antigen receptors. It is believed that it will be easier to control the function of the next generation CAR-T cells and thereby minimizing their side effects [94].

## CAR-T cells in veterinary oncology

ACT therapy in dogs is not as advanced as in humans and therefore the literature on the subject is still limited. To date, just two groups have attempted to use CAR technology to treat canine osteosarcoma (OS) and B cell lymphoma. These tumors are considered as valuable model of human cancers as they share similar tumor-associated antigens such as HER2 (osteosarcoma associated antigen) and CD20 (marker of transformed B cells).

The ACT study to treat osteosarcoma was performed with T cells obtained from peripheral blood of healthy dogs [65]. The T lymphocytes were activated using irradiated K562 aAPCs, genetically modified to express human CD80, CD83, CD86, 41BBL. Additionally, cells were stimulated with phytohemagglutinin (PHA) and rhIL-21. Interestingly, other methods of activation, such as administration of Concanavalin A (ConA) or anti-human CD3 (OKT-3 clone) antibodies coated-plates were insufficient to effectively stimulate canine T cell proliferation for ACT therapy purpose [65, 95]. Activated T cells were subsequently transfected with a 2nd generation α-canine HER2 CAR (containing CD3ζ and CD28 domains). The authors demonstrated that HER2-CAR-T cells in co-culture with several HER2 positive canine OS cell lines secreted more IFN-γ and exhibited superior ability to eliminate HER2^+^ OS cells in vitro. The results were not observed in HER2^−^ OS-phenotype cell lines or non-transfected T cells. Despite these promising results, this approach has not yet been evaluated in vivo.

Building on these encouraging in vitro data, Panjwani et al. [95] performed in vivo studies on dogs suffering from B cell lymphoma. Autologous canine T cells were transiently transfected with α-canine CD20 CAR using electroporation. In the presence of canine lymphoma cells the CD20-CAR-T cells secreted significantly more of IFN-γ, than non-modified T cells or CD19-CAR-T cells (used as non-specific CAR transfection control). Additionally, antigen-specific CAR-T cells actively lysed the target cancer cells. Dogs with B cell lymphoma that relapsed after chemotherapy (l-asparaginase, vincristine, cyclophosphamide, doxorubicin or prednisone) were given three doses of 700,000 CD20-CAR-T cells per kg. Serious adverse side effects were not observed. Each injection resulted in an enlargement of the target lymph node, decreased frequency of cancer CD79a^+^/CD20^+^ cells and increase of non-transformed CD5^+^ B lymphocytes in the lymph nodes. In addition, increased serum levels of IL-6 and IFN-γ were measured after the first dose of CD20-CAR-T cells [95]. Unfortunately, due to the transient transfection of these CAR-T cells, durable remission was not attained.

## Lymphokine-activated killer (LAK) cells for adoptive cell therapy

In contrast to adoptive T cell transfer therapy which exploits tumor-specific T cells, passive immunotherapy involves administration of autologous lymphocytes, without cancer specificity. In human medicine, this type of immunotherapy is referred to as lymphokine-activated killer (LAK) cell therapy and was among one of the first cell transfers [96] discarded in a favor of newer technologies (ACT therapy using TCR- or CAR-engineered T cells). However, LAK cells transfer was preferentially investigated in veterinary medicine, due to the low cost of cell generation. LAK cells are derived from PBMC stimulated with α-CD3 antibody-coated plates and soluble hrIL-2. Initially, LAK cell therapy was tested in healthy beagles [97]. Sequential administration of LAK cells increased host immune cell proliferation and IFN-γ levels in the serum without inducing severe side effects. These results suggested that LAK cell therapy is safe and could promote an immune response. Several papers described LAK cells’ generation and anticancer activity against canine thyroid carcinoma and melanoma cell lines in vitro [97–99]. Recently, LAK cell therapy, in combination with surgery, was evaluated in vivo in 15 tumor-bearing dogs [100]. Patients received five rounds of LAK cell transfer with 2–4 weeks intervals. Single transfer resulted in an increase of CD8^+^ T cells’ frequency and a decreased ratio of CD4^+^ to CD8^+^ T cells. Although the immunoenhancing effect of LAK cell transfer was confirmed, this type of immunotherapy alone has not mediated potent anticancer immune response or complete tumor eradication. Therefore, LAK cell transfer is not suitable as a monotherapy, but its application is promising as an adjuvant treatment.

A summary of different immunotherapeutic approaches in veterinary medicine have been compiled in Table 2.

**Table 2:** Adoptive cell transfer immunotherapies in veterinary medicine

Therapy type	Attempts to use in veterinary oncology	
TIL therapy	Non-Hodkgin’s lymphoma	In vivo [64]
CAR-T cell transfer	Osteosarcoma	In vitro [65]
	B-cell lymphoma	In vitro and in vivo [95]
T-LAK cell transfer	Thyroid cancer	In vitro and in vivo [98, 100]
	Melanoma	In vitro and in vivo [98, 100]
	Hepatocarcinoma	In vivo [100]
	Fibrosarcoma	In vivo [100]

## Exploiting genome-editing technology to improve T cell phenotype

Genetic engineering technology is used for modifying of both TCR- and CAR-engineered T cells. Novel approaches focused on modulation of chemokine receptor expression and cytokine secretion of T lymphocytes or resistance to immunosuppressive tumor microenvironment by effector cells.

**Fig. 4:**
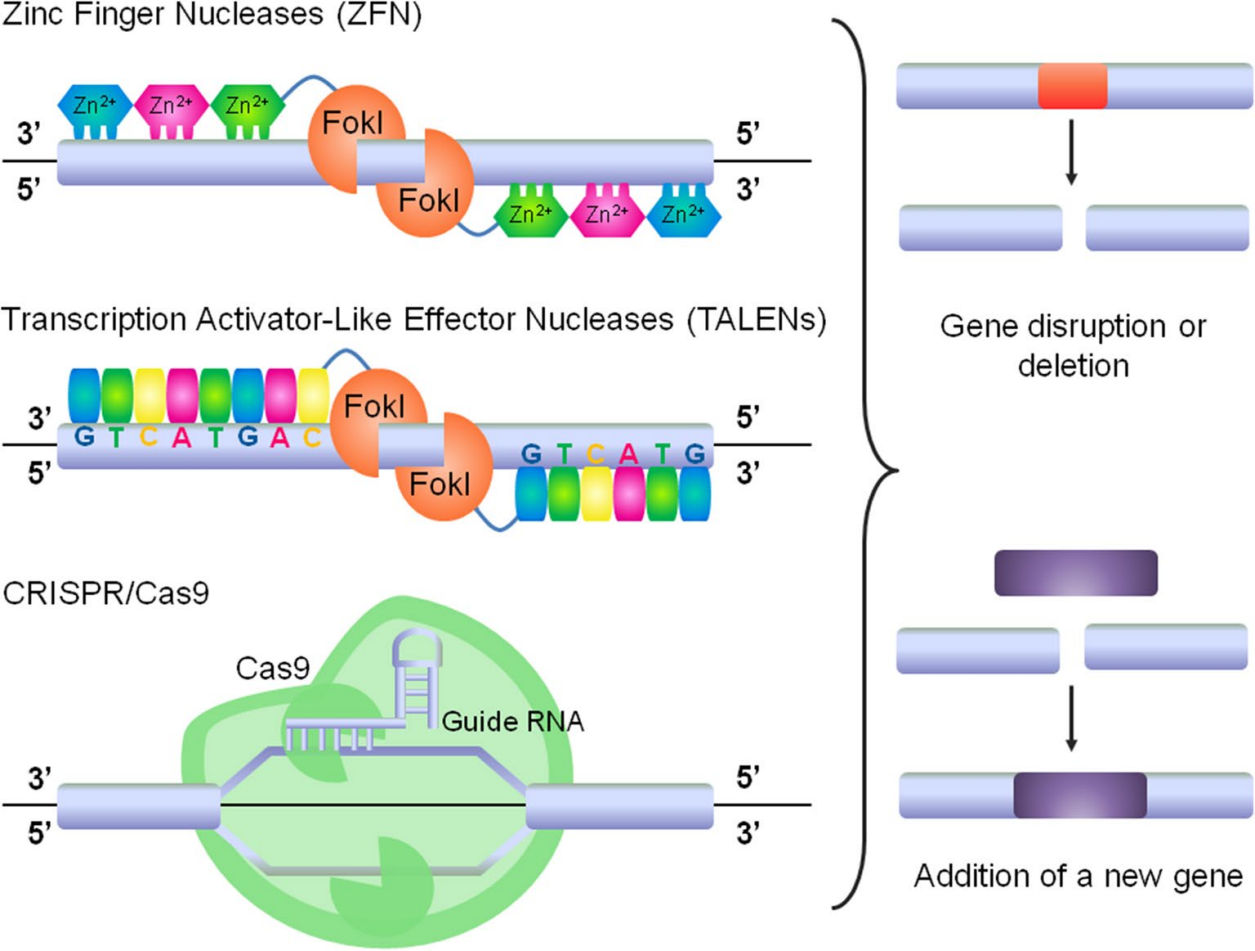
Genome editing systems are used to introduce double-strand breaks into DNA, which allow for gene correction, deletion or addition ZFNs, TALENs and CRISPR/Cas9 are versatile nuclease-based platforms of genome editing technology. ZFNs and TALENs consist of DNA-binding domain, which is engineered to recognize specific sequences and nuclease domain—FokI—responsible for DNA cleavage. CRISPR/Cas9 is based on RNA-guided DNA recognition complex which interacts with Cas9 nuclease catalyzing site-specific breaks in DNA. Genome editing technologies can be used for the immunotherapy purposes to enhance T cells properties

To date, three methods of genome editing have been implemented in the clinic: (1) zinc finger nucleases (ZFN), (2) transcription activator-like effector nuclease (TALEN) and the most recent (3) clustered regularly interspaced short palindromic repeats (CRISPR) associated protein system (CRISPR/Cas9) (Fig. 4) [101]. ZFN and TALEN are chimeric nucleases that contain a DNA-binding domain (engineered to recognize specific sequences) and a DNA cleavage enzyme (usually FokI) [102–104]. CRISPR/Cas9 system is based on RNA-guided DNA sequence recognition followed by cleavage by Cas9 endonuclease [105]. Each of these methods introduces nuclease-induced DNA double strand breaks into the sequence of interest. Importantly, these technologies not only improve gene silencing, but also allows for other genetic manipulations such as gene correction, deletion or insertion (Fig. 4) [106]. Thus, they appear to be a promising and useful tool for biomedical research.

The first in vivo success of this technology was achieved using ZFN to treat haemophilia in a murine model-correcting this genetic disorder and restorating normal blood coagulation [107]. Genome-editing technologies were also employed for treating of hereditary tyrosinemia (HTI), Duchenne muscular dystrophy (DMD) and hepatitis B virus (HBV) infection [108–110].

Recently, T cells modified using TALEN-based technology were transferred to leukemia patients [111]. Allogeneic healthy donor T cells were genetically engineered to prevent graft-versus-host disease (GVHD) upon adoptive cell transfer. Infused T cells did not cause an immune response in the recipient. This technique has been used just to prolong a patients’ survival time until suitable donor is found. In followup studies, CD19 CAR-T cells with disrupted TCR and CD52 coding genes were used to effectively treat B cell malignancies in infant [112]. Authors speculated that due to the lack of TCR expression, heterologous CAR-T cells can potentially be utilized for every patient with B cell leukemia [112, 113]. These study confirmed that gene editing technology may help to solve problems associated with engineering T cells. Similarly, ZFN-mediated disruption of the endogenous TCR genes in WT1 antigen-specific T cells prevented GVHD upon transfer into mice. This procedure prevents TCR heterodimmer formation, which is one of the major drawbacks of TCR-engineered T cell transfer therapy [114]. Recent advances in genetic manipulations can allowed to reduce adverse effects of ACT therapy and make it more widely applicable for the treatment for a range of malignancies. However, there are still several limitations of using genome editing technologies, which comprise: low efficiency of the method in some cell types, construct delivery problems, single-instead of double-stranded breaks, and off-target effects of nucleases and genetically modified cells (Table 1) [106, 115].

Genome editing has not been fully exploited in canine patients. Nevertheless, CRISPR/Cas9 method has been used to generate beagles with a myostatin gene knockout [116]. The study proved the feasibility of canine genome editing technique and has paved the way to generate other knockout dogs, potentially serving as models of multiple disease in the biomedical sciences.

## Conclusion

In the past few years, we have witnessed amazing successes in harnessing immune system to fight against cancer. The American Society of Clinical Oncology selected cancer immunotherapy as the 2016 “Advance of the Year” for its unprecedented effectiveness in eradicating various types of tumors. In the future, more efforts should be made to utilize translational veterinary medicine to benefit human health. The domestic dog model offer multiple advantages to move cancer immunotherapies forward. Most importantly, these treatments may be also the silver lining in veterinary oncology giving hope for canine patients.

## Abbreviations

aAPC: artificial antigen presenting cells
ACT: adoptive cell transfer
ADCC: antibody-dependent cellular cytotoxicity
CAR: chimeric antigen receptor
CCL-20: chemokine (C-C motif) ligand 20
CCR5: chemokine receptor 5
CCR7: C-C chemokine receptor type 7
CD: cluster of differentiation
ConA: concanavalin A
CRISPR/Cas9: clustered regularly interspaced short palindromic repeats (CRISPR) associated protein system with Cas9 endonuclease
CTLA-4: cytotoxic T cell antigen 4
CTLs: cytotoxic T lymphocytes, CD8^+^ positive cells
DC: dendritic cells
DLA: dog leukocyte antigen
DMD: Duchenne muscular dystrophy
GVHD: graft-versus-host disease
HBV: hepatitis B virus
HER2: human epidermal growth factor receptor 2
HLA: human leukocyte antigen
HTI: hereditary tyrosinemia I
ICOS: inducible T cell costimulator
IFN-γ: interferon-γ
IL: interleukin
LAK: lymphokine-activated killer cells
MART1: melanoma antigen recognized by T cells 1
MDSC: myeloid-derived suppressor cells
MHC: major histocompatibility complex
NK cells: natural killer cells
NKT cells: natural killer T cells
NY-ESO-1: cancer–testis antigen
OS: canine osteosarcoma
PD-1: programmed cell death protein 1
PHA: phytohemagglutinin
RCC: renal cell carcinoma antigen
rhIL: recombinant human interleukin
ROR: retinoic acid-related orphan receptor
scFv: single chain variable fragment
TALEN: transcription activator-like effector nuclease
TAMs: tumor-associated macrophages
TCR: T cell receptor
TGF-β: tumor growth factor β
Th cells: helper CD4^+^ T cells
TILs: tumor infiltrating lymphocytes
TNF-α: tumor necrosis factor α
Tregs: T regulatory cells
WT1: Wilms tumor antigen-1
ZFN: zinc finger nucleases
